# Neutrophils as key drivers of pulmonary fibrosis: unveiling mechanisms and therapeutic implications

**DOI:** 10.3389/fimmu.2025.1718092

**Published:** 2025-11-27

**Authors:** Xiuping Liang, Yanhong Li, Ziyi Tang, Yubin Luo, Yi Liu

**Affiliations:** 1 Department of Rheumatology & Immunology, Laboratory of Rheumatology and Immunology, West China Hospital, Sichuan University, Chengdu, Sichuan, China; 2 West China Lecheng Hospital, Sichuan University, Boao, Hainan, China

**Keywords:** pulmonary fibrosis, neutrophils, neutrophil extracellular traps, neutrophil elastase, crosstalk

## Abstract

Pulmonary fibrosis is a chronic interstitial lung disease with an incompletely understood pathogenesis, and currently, effective treatment strategies remain elusive. Neutrophils, as pivotal effector cells of the innate immune system, are integral to the progression of pulmonary fibrosis. This review systematically examines the mechanisms by which neutrophils contribute to the advancement of pulmonary fibrosis through tissue infiltration, the release of neutrophil elastase (NE), and the formation of neutrophil extracellular traps (NETs). The interactions between neutrophils and other cell types, including alveolar macrophages, epithelial cells, and fibroblasts, create a complex inflammatory and fibrotic network. Clinical studies suggest that neutrophil levels and associated biomarkers, such as NET components, may serve as valuable indicators for disease assessment. Targeted therapeutic strategies, such as NE inhibitors, peptidyl arginine deiminase 4 (PAD4) inhibitors, blockade of the C5a-C5aR1 axis, and stem cell therapy, present promising avenues for the treatment of pulmonary fibrosis. This article aims to provide a comprehensive overview of the multifaceted roles of neutrophils in pulmonary fibrosis and their therapeutic implications.

## 1 Introduction

Pulmonary fibrosis (PF) is a chronic and often fatal condition marked by the thickening of alveolar walls, excessive extracellular matrix (ECM) deposition, disruption of lung architecture, and progressive respiratory failure (1). The disease presents highly heterogeneous trajectories and is associated with elevated mortality rates (2). The etiological factors of PF are varied, encompassing environmental exposures (3–5), viral infections (6), genetic predispositions (7) and connective tissue disorders (8) etc. While certain pharmacological interventions, such as pirfenidone and nintedanib, can decelerate the deterioration of lung function (9), there is a notable absence of curative therapies. This underscores the urgent necessity for a more comprehensive understanding of the underlying disease mechanisms (2, 9). The fundamental pathological processes involve epithelial injury (10), dysregulated immune cell activation (11–15), and persistent fibroblast activation (16), culminating in irreversible ECM accumulation.

**Figure 1:**
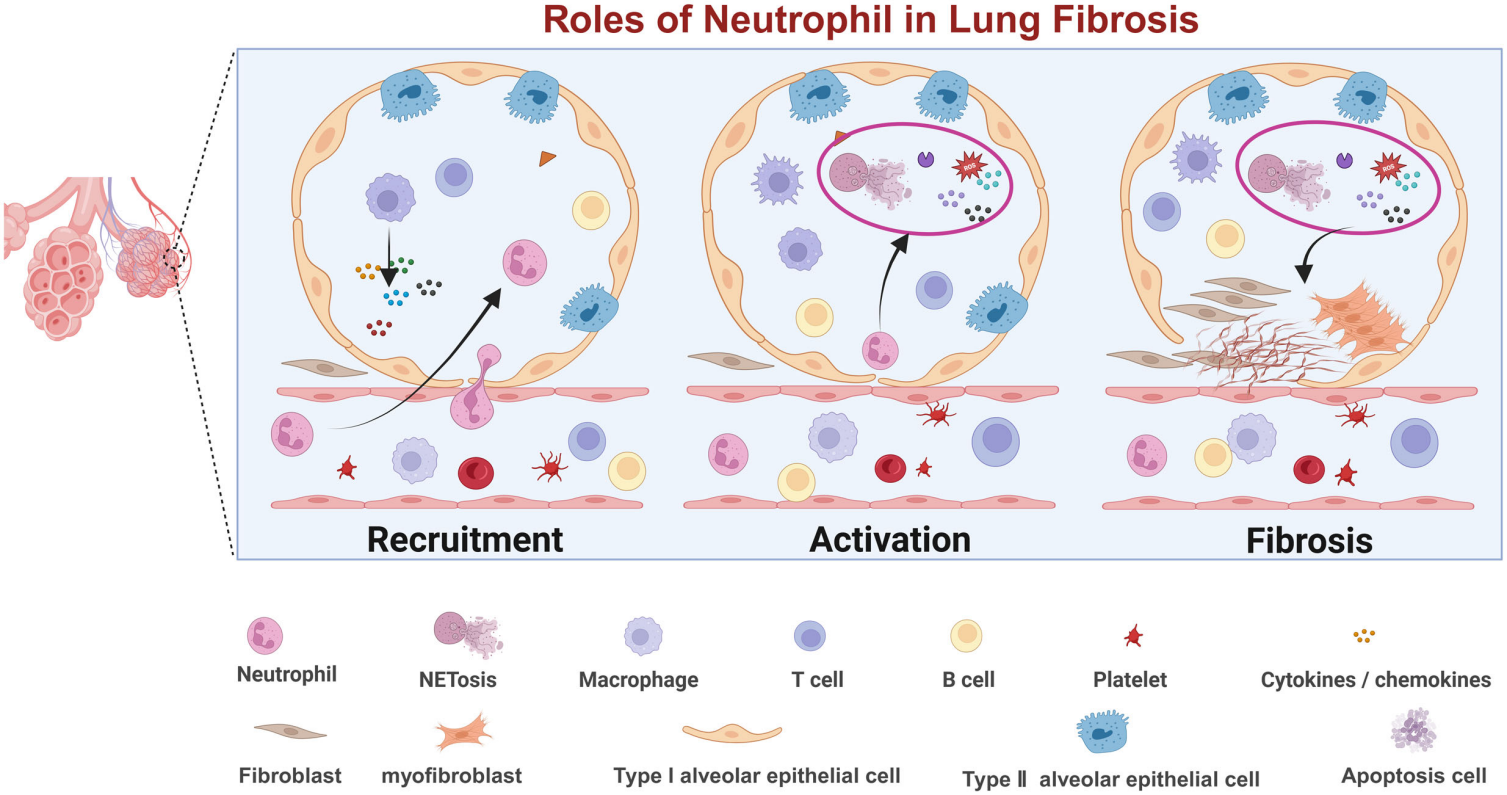
Diagram illustrating the roles of neutrophils in lung fibrosis across three stages: recruitment, activation, and fibrosis. https://app.biorender.com.

In the early stages of pulmonary fibrosis, chronic lung injury or external stimuli, such as exposure to fine particulate matter or dust irritation, can result in damage and activation of alveolar epithelial cells. This process initiates inflammatory signaling pathways and promotes the release of various inflammatory factors, including NOD-like receptor family, pyrin domain containing 3 (NLRP3) and interleukin-1 beta (IL-1β) (5, 17). The ensuing inflammatory response facilitates the recruitment of immune cells, such as neutrophils, macrophages, and monocytes, into the lung tissue, leading to local immune dysregulation and the establishment of a persistent inflammatory microenvironment (18, 19). Neutrophils, as early responders, are swiftly recruited to the injury site, where they release proteases, such as elastase, and reactive oxygen species (ROS), thereby amplifying the inflammatory response. Furthermore, they secrete pro-inflammatory mediators, including interleukin-6 (IL-6) and interleukin-8 (IL-8), which enhance local oxidative stress and compromise the epithelial barrier, thus exacerbating the propagation of inflammation (15). Macrophages, including monocyte-derived interstitial types, infiltrate and replace alveolar macrophages, releasing pro-fibrotic mediators like transforming growth factor-β1 (TGF-β1) and Tumor Necrosis Factor-α (TNF-α), which maintain inflammation and boost collagen production and pro-fibrotic signaling (14, 20). Immune cell infiltration triggers a pro-fibrotic response that drives fibroblasts to transdifferentiate into myofibroblasts. For example, TGF-β1 from macrophages activates the Smad pathway in fibroblasts, increasing α-smooth muscle actin (α-SMA) expression and enhancing myofibroblast contractility and migration (21). Additionally, neutrophil-derived proteases can enhance TGF-β1 signaling, contributing to excessive myofibroblast differentiation and ECM deposition (15). These processes result in increased ECM components, matrix stiffening, and ultimately, irreversible fibrotic scars that drive progressive pulmonary fibrosis (**Figure 1**).

Within the context of the immune-inflammatory mechanisms underlying PF, neutrophils—recognized as the most prevalent immune cells in humans (15, 22)—play a pivotal role through their recruitment and activation within the airways. Originating from the bone marrow, neutrophils are mobilized to sites of inflammation in response to infection or tissue injury, where they engage in phagocytosis, exhibit bactericidal activity, and release proteases such as elastase through degranulation. Recent investigations have demonstrated markedly increased neutrophil levels in both the airways and circulation of patients with interstitial lung disease (ILD), particularly idiopathic pulmonary fibrosis (IPF), with these levels showing a positive correlation with disease severity and mortality (23). Beyond their traditional inflammatory functions, neutrophils contribute to the progression of PF through the formation of neutrophil extracellular traps (NETs), which facilitate the release of pro-fibrotic mediators (e.g., proteases) (24), activate TGF-β1 signaling pathways, and induce damage to alveolar epithelial cells (24).

A comprehensive understanding of the regulatory networks mediated by neutrophils in PF is of dual importance: firstly, neutrophil counts and levels of NETs may serve as prognostic biomarkers, such as the neutrophil-lymphocyte ratio (NLR) (25); secondly, targeting neutrophil-associated pathways—such as inhibiting NETs formation (24), blocking complement component 5a receptor 1(C5aR1) signaling—offers potential as an innovative therapeutic strategy (26). This review systematically synthesizes the molecular mechanisms through which neutrophils contribute to pulmonary fibrosis, encompassing the roles of proteolytic enzymes, NET-mediated fibrotic signaling, and microenvironmental regulation. Furthermore, it assesses the therapeutic potential of strategies targeting neutrophils in reversing fibrotic progression, thereby providing a theoretical framework to address current clinical challenges.

## 2 Neutrophil pro-fibrotic functions

Neutrophils represent the most prevalent leukocyte population in human peripheral blood and are integral components of the innate immune system. They are crucial for host defense, the regulation of inflammation, and the pathogenesis of various diseases (27, 28). Derived from hematopoietic stem cells within the bone marrow, neutrophils undergo a process of granulopoiesis, culminating in their maturation into terminally differentiated cells (29). Upon maturation, these cells are swiftly released into the circulatory system, where they have a brief lifespan (30). They are subsequently removed through apoptosis and clearance mechanisms such as phagocytosis, or via reverse migration from sites of inflammation, thus playing a role in maintaining tissue homeostasis (31, 32). The recruitment of neutrophils to sites of infection or injury is mediated by chemotactic factors, which promote their rapid migration from the bloodstream into tissues through processes involving diapedesis and chemotaxis (33). Neutrophils perform a variety of effector functions through well-coordinated mechanisms (30).

### 2.1 Neutrophil chemotaxis

Neutrophil chemotaxis constitutes the initial phase of their physiological and pathological roles. This directed migration is predominantly stimulated by chemokines such as C-X-C motif chemokine ligand (CXCL) 1, CXCL2, CXCL6, CXCL16, interleukin (IL)-8, and interleukin-36γ (IL-36γ) (34). These chemokines engage with specific receptors on the neutrophil surface, triggering downstream signaling pathways that direct cellular migration (35, 36). CXCR1 functions as the receptor for CXCL6 and IL-8, whereas CXCR2 binds to CXCL1, CXCL2, CXCL6, and IL-8 (37, 38). Under physiological conditions, neutrophils adeptly detect chemotactic gradients, such as the bacterial peptide N-formylmethionyl-leucyl-phenylalanine (fMLP), and migrate to sites of inflammation through directed migration, biased random walk, and front-rear coordination, thereby facilitating effective pathogen clearance (39–41). Neutrophils play a crucial role in the initiation and progression of fibrosis across various organs through their chemotactic capabilities. In a corneal fibrosis model induced by alkali burns, leucine-rich alpha-2-glycoprotein 1 (LRG1) enhances neutrophil chemotaxis and modulates the IL-6/STAT3 signaling pathway to drive the fibrotic response in the cornea (42). Conversely, the application of LRG1-specific small interfering RNA leads to a reduction in the expression of fibrotic proteins and neutrophil infiltration in this model (42). In the context of non-alcoholic steatohepatitis (NASH), IL-8 targets CXCR2 to facilitate neutrophil infiltration and activation, thereby promoting fibrotic progression (43). The formyl peptide receptor 1 (FPR1) expressed on neutrophils is instrumental in their recruitment into pulmonary fibrotic tissues, and a deficiency in FPR1 has been shown to confer protection against the development of pulmonary fibrosis (44).

### 2.2 Neutrophil phagocytosis and degranulation

Neutrophil phagocytosis represents a critical mechanism for the elimination of pathogens. This process is initiated by the internalization of particles into phagosomes, followed by their degradation through a burst of ROS facilitated by nicotinamide adenine dinucleotide phosphate (NADPH) oxidase, along with the coordinated release of granule enzymes (45, 46). Under physiological conditions, the functionality of neutrophils displays significant heterogeneity both inter-individually and at the single-cell level s (47). This variability is influenced by gene expression profiles and intrinsic signaling pathways, such as those mediated by Fcγ receptors, including FcγRIIa (48).

The process of neutrophil degranulation operates in conjunction with phagocytosis as a critical immune mechanism. This process entails the release of various granule constituents, such as myeloperoxidase (MPO), neutrophil elastase (NE), and matrix metalloproteinases (MMP-8/9) (49). The initiation of degranulation occurs via Syk-dependent signaling pathways (50) and can be enhanced by the extracellular signal-regulated kinase 1/2 (ERK1/2) pathway (51). From a physiological perspective, degranulation contributes to pathogen clearance through the release of antimicrobial proteins and acts synergistically with ROS generation and phagocytosis to resolve infections (46).

The dysregulation of these functions plays a pivotal role in tissue injury and the pathogenesis of various diseases. In cystic fibrosis (CF), the airways are infiltrated by a specific subset of neutrophils known as GRIM (granule-releasing, immunomodulatory, and metabolically active), which demonstrate impaired bacterial phagocytosis, which impairment significantly increases patients’ susceptibility to common environmental bacteria (52). Additionally, these neutrophils, when stimulated by granulocyte colony-stimulating factor (G-CSF) and granulocyte-macrophage colony-stimulating factor (GM-CSF), excessively release NE and MMP-9, thereby exacerbating airway damage (53). In IPF, elevated serum levels of copper-zinc superoxide dismutase, potentially released through degranulation or from damaged neutrophils, are associated with disease severity and may contribute to free radical-mediated tissue injury and fibrosis (54). In the context of atrial fibrillation, neutrophils infiltrating the atrial epicardial adipose tissue secrete substantial amounts of MPO, which directly activates atrial fibroblasts and induces pro-fibrotic responses (55).

### 2.3 NETosis

NETosis represents a distinct form of neutrophil cell death primarily induced by ROS and NADPH oxidase (56). This process involves chromatin decondensation and histone citrullination, culminating in the release of NETs (56, 57). Upon activation, neutrophils generate ROS through NADPH oxidase, which subsequently activates MPO and NE, leading to membrane rupture and the release of DNA (58). NETosis can occur in both NADPH-dependent (“suicidal”) and independent (“vital”) forms, ultimately resulting in NETs formation and cell death (58). NETs are composed of DNA scaffolds, histones, and antimicrobial proteins such as NE and MPO, which facilitate the trapping and eradication of pathogens (59). Physiologically, NETosis enhances innate immunity by capturing and neutralizing microbes, thereby controlling infections and modulating inflammatory responses (60, 61).

NETosis plays a crucial role in the fibrotic processes across various organs. NETs contribute to the pathogenesis of fibrosis by perpetuating inflammation and causing tissue damage. In the context of liver fibrosis, NETs exacerbate hepatic tissue injury and promote abnormal collagen deposition (62). In pulmonary fibrosis, NETs significantly accelerate the formation of lung scars and impair lung function by activating TGF-β1 and inducing damage to alveolar epithelial cells (15). In the renal domain, patients with IgA nephropathy exhibit elevated levels of NETosis markers, such as citrullinated histone H3 and myeloperoxidase, which correlate with the severity of glomerular fibrosis and facilitate collagen deposition by stimulating mesangial cells (63). In systemic sclerosis-associated skin fibrosis, there is a marked increase in NET production during the early stages of the disease, which is linked to excessive skin collagen accumulation (64). In cardiac fibrosis, NETosis mediates the myocardial inflammatory response through neutrophil-specific enzymes, such as myeloperoxidase, and contributes to fibrotic remodeling during heart failure (65). In short, NETosis is key in fibrosis development across various organs by inducing chronic inflammation and NET release, linking inflammation with tissue fibrosis.

### 2.4 Neutrophil-derived pro-fibrotic mediators

Neutrophils are capable of producing and releasing a variety of pro-fibrotic cytokines and chemokines, including IL-1β, IL-6, TNF-α, CCL3, IL-8, CXCL1, and CXCL2, which facilitate the initiation and progression of fibrosis (66, 67). The simultaneous retention of Mincle-positive neutrophils and macrophages during the transition from acute kidney injury to chronic kidney disease results in persistent inflammation, thereby promoting fibrosis, with TNF-α serving as a pivotal pro-inflammatory cytokine (68). IL-6 contributes to fibrotic progression through STAT3-mediated fibroblast senescence (69), while IL-1β-driven macrophage activation and tubular cell senescence further exacerbate renal fibrosis (70). Furthermore, In animal studies, high IL-23 levels are strongly linked to increased neutrophil infiltration and worsening lung structure, which promotes neutrophilic inflammation during acute exacerbations of idiopathic pulmonary fibrosis (IPF) and may indicate poor prognosis (71).

## 3 Neutrophil-centric crosstalk in lung fibrosis

**Figure 2:**
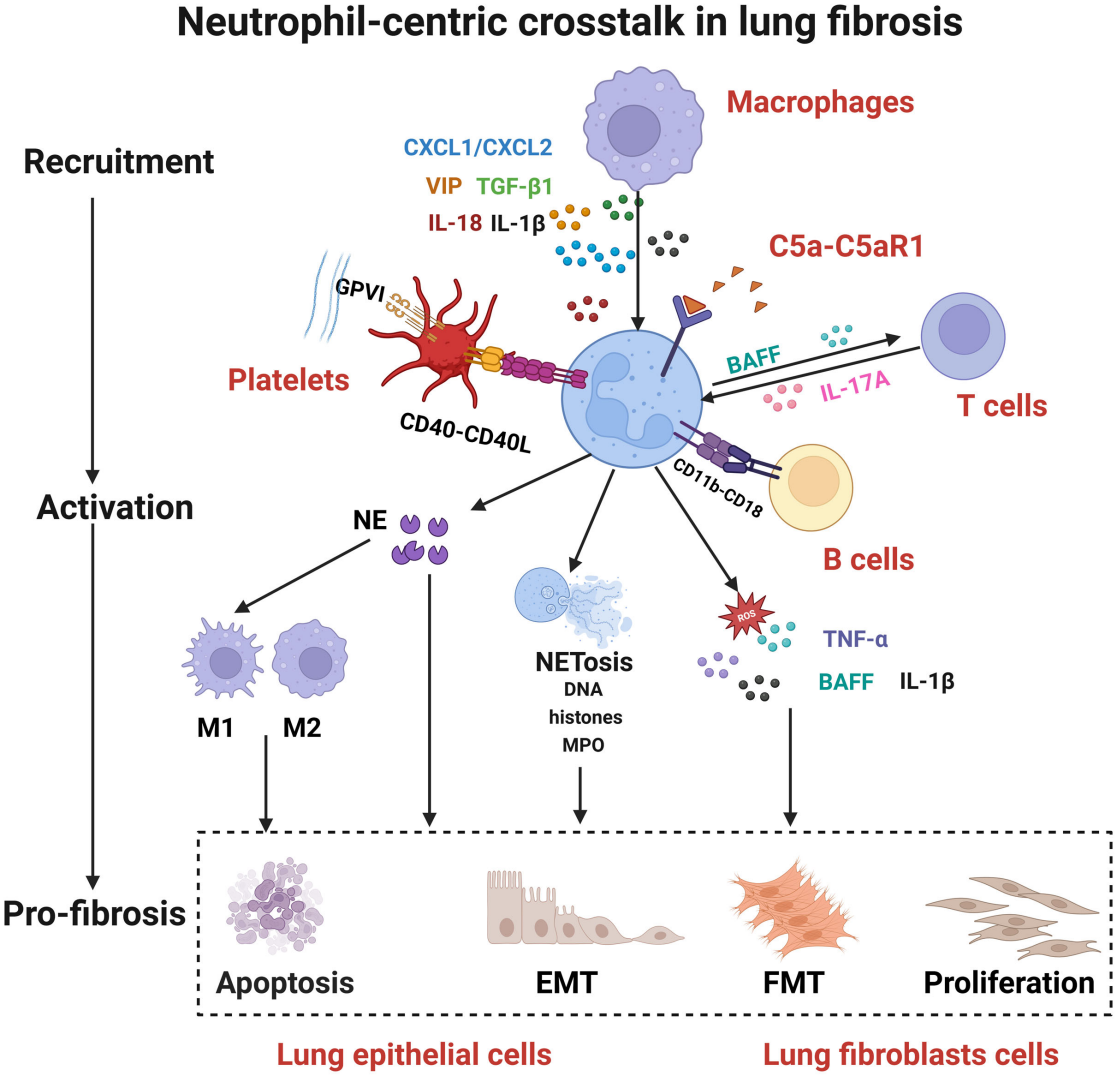
Neutrophil-centric crosstalk in lung fibrosis. The fibrotic process involves coordinated cellular interactions: (1) Macrophages, T cells, and platelets recruit and activate neutrophils; (2) Activated neutrophils release NETs, NE, IL-1β, and TNF-α; (3) These effector molecules target lung epithelial and fibroblast cells, promoting apoptosis, EMT, and FMT to drive fibrosis. CXCL2, C-X-C motif chemokine ligand 2; VIP, vasoactive intestinal peptide; TGF-β1, transforming growth factor beta 1; IL-18, interleukin-18; IL-1β, interleukin-1 beta; C5a, complement component 5a; C5aR1, complement C5a receptor 1; GPV, glycoprotein V; CD40L, CD40 ligand; BAFF, B cell activating factor; IL-17A, interleukin-17A; NETosis, neutrophil extracellular trap formation; DNA, deoxyribonucleic acid; TNF-α, tumor necrosis factor alpha; IL-1β, interleukin-1 beta; MPO, myeloperoxidase; NE, neutrophil elastase; EMT, epithelial-mesenchymal transition; FMT, fibroblast-myofibroblast transition. https://app.biorender.com.

Neutrophils are pivotal cells implicated in the initiation and progression of PF. Their pro-inflammatory and pro-fibrotic roles are contingent upon interactions with a diverse array of immune and tissue cells. To comprehensively elucidate the contribution of neutrophils to pulmonary fibrosis, it is imperative to delineate the intricate interactions between neutrophils and these cellular counterparts. In the subsequent section, we will conduct an in-depth examination of the mechanisms through which neutrophils engage with neighboring cells in the lung—namely macrophages, lymphocytes, fibroblasts, and epithelial cells—and collectively facilitate the progression of the disease (**Table 1**, **Figure 2**).

**Table 1:** Neutrophil-centric crosstalk in lung fibrosis.

Interacting cell	Model	Mechanism (detailed)	Ref.
Macrophage	Silica-induced mice	Macrophage NLRP3 inflammasome activation promotes IL-1β/IL-18 releases, driving neutrophil infiltration and NE activation, leading to TGF-β-mediated fibrosis.	2025 (72)
	PM-induced mice	Macrophage-derived KC (CXCL1) is a key chemokine for recruiting neutrophils into the lungs.	2019 (73)
	BLM-induced mice	Loss of neuronal signaling causes alveolar macrophages to produce VIP, which induces TGF-β1 production and the expansion of a pro-fibrotic Siglec-F+ neutrophil subset.	2025 (74)
	BLM-induced mice	Macrophages in fibrotic lungs upregulate CXCL2 expression, mediating the sustained recruitment of neutrophils.	2022 (75)
	*In vitro* (THP-1 cells)	NETs from stimulated neutrophils are phagocytosed by macrophages, causing oxidative stress, mitochondrial dysfunction, and driving polarization toward a pro-fibrotic M1/M2 phenotype.	2025 (12)
Platelets	HOCl/BLM-induced SSc mice	Platelet activation via the GPVI collagen receptor triggers neutrophil activation and NETosis. NETs are identified as key effector molecules driving tissue fibrosis.	2025 (81)
	BLM-induced mice	CD40-CD40L interaction mediates platelet-neutrophil adhesion. Inhibition of platelet activation (by cangrelor) reduces neutrophil infiltration and fibrosis.	2020 (82)
T cells (IL-17A^+^)	BLM-induced mice	Gr1^+^ neutrophils produce BAFF under IL-1β/IL-17A induction. BAFF then acts on IL-17A^+^ T cells to amplify IL-17A signaling, creating a positive feedback loop that promotes fibrosis.	2015 (88)
	BLM-induced mice	IL-17A+ γδ T cells enhance neutrophil infiltration and shift macrophages to the M2 phenotype in the lungs, speeding up fibrosis.	2016 (90)
B cells	CD19-DTR mice Rosa26-DTR mice	B cells engage with senescent neutrophils through β integrins (CD11b–CD18) to promote their apoptosis and clearance, preventing excess neutrophils and reducing inflammation and fibrosis. The CXCR4 antagonist AMD3100 decreases B cell and neutrophil presence in the lungs, directly slowing pulmonary fibrosis progression.	2018 (91)
Complement system	SWCNT-induced mice	Activation of the C5a-C5aR1 signaling axis promotes early neutrophil recruitment, TNF-α/IL-1β release, and subsequent fibrosis. A C5aR1 antagonist (PMX205) inhibits this process.	2025 (26)
Lung epithelial cell	BBP-induced mice	BBP induces metabolic reprogramming in neutrophils (increased glucose uptake & ROS burst), leading to NETosis. NETs then directly drive fibrotic transformation of epithelial cells.	2022 (124)
	BLM induced rats	NE induces apoptosis via activating caspase-3/9 and cytochrome c release. Sivelestat inhibits this process.	2009 (92)
	PM-induced mice	NE released from neutrophils promotes EMT and fibrosis via macrophage-derived KC and SMAD2/3/α-SMA pathway.	2019 (73)
	Severe COVID-19 patients & airway *in vitro* model	NETs cooperate with AM-derived factors (TGF-β, IL-8, IL-1β) to induce EMT (↓E-cadherin, ↑α-SMA).	2021 (93)
	Patients with SLE and COVID-19-related PF	NETs promote EMT (↑Twist, Snail, α-SMA; ↓E-cadherin) via a common transcriptomic pathway.	2023 (94)
Lung fibroblast	Patients with NSIP; *in vitro* NET stimulation	NET components (DNA, histones, MPO) induce myofibroblast differentiation; NET-derived IL-17 upregulates CCN2 and collagen.	2014 (96)
	Asbestos-induced mice	NE directly promotes fibroblast proliferation and myofibroblast differentiation (α-SMA expression) in a TGF-β-independent manner.	2015 (95)
	BLM-induced mice	PAD4-dependent NETosis promotes fibroblast activation and fibrosis; rescued by PAD4 deficiency.	2020 (98)
	Particulate Matter (PM)-induced mouse model	NETs activate fibroblasts and promote fibrosis via the TLR9–miR-7–SMAD2 pathway.	2020 (97)
Lung fibroblast and epithelial cell	MAILD model	NETs induce EMT and NLRP3 inflammasome activation; Pirfenidone inhibits NETosis and alleviates fibrosis.	2025 (99)

COVID, corona virus disease; NETs, neutrophil extracellular traps; NE, neutrophil elastase; NLRP3, NOD-like receptor family, pyrin domain containing 3; IL-1β, interleukin-1 beta; IL-18, interleukin-18; TGF-β, transforming growth factor-beta; KC, keratinocyte chemoattractant; CXCL1, C-X-C motif chemokine ligand 1; PM, particulate matter; VIP, vasoactive intestinal peptide; CXCL2, C-X-C motif chemokine ligand 2; THP-1, human acute monocytic leukemia cell line; HOCl, hypochlorous acid; SSc, systemic sclerosis; GPVI, glycoprotein VI; CD40-CD40L, CD40 - CD40 ligand; BALF, bronchoalveolar lavage fluid; IL-17A, interleukin-17A; SWCNT, single-walled carbon nanotube; C5a-C5aR1, complement component 5a - c5a receptor 1; TNF-α, tumor necrosis factor-alpha; BBP, benzyl butyl phthalate; BLM, bleomycin; α-SMA, alpha-smooth muscle actin; EMT, epithelial-mesenchymal transition; AM, alveolar macrophage; SLE, systemic lupus erythematosus; PF, pulmonary fibrosis; NSIP, Nonspecific Interstitial Pneumonia; MPO, myeloperoxidase; CNN2, calponin 2; PAD4, peptidyl arginine deiminase 4; TLR9, toll-like receptor 9; MAILD, murine myositis-associated interstitial lung disease; Ref., reference.

### 3.1 Crosstalk between neutrophils and other immune cells

#### 3.1.1 Macrophage

A complex bidirectional regulatory relationship exists between macrophages and neutrophils. Firstly, macrophages serve as critical upstream regulators to recruit and activate neutrophils. In the context of silica-induced pulmonary fibrosis, the activation of the NLRP3 inflammasome in alveolar macrophages and other lung cells facilitates the release of IL-1β and IL-18. This process drives neutrophil infiltration into the airways and activates NE, ultimately resulting in TGF-β-mediated myofibroblast activation and fibrosis (72). Following exposure to particulate matter (PM), keratinocyte chemoattractant (KC) produced by macrophages functions as a key chemokine for neutrophil recruitment (73). In the bleomycin (BLM) model, the loss of vagal sensory neurons prompts alveolar macrophages to produce vasoactive intestinal peptide (VIP), which induces TGF-β1 production and promotes the accumulation of a pro-fibrotic Siglec-F^+^ neutrophil subset (74). Additionally, macrophages in fibrotic lungs exhibit high expression levels of CXCL2, facilitating sustained neutrophil recruitment (75). Conversely, activated neutrophils and NETs influence macrophage polarization. Studies show that when macrophages engulf NETs, they experience oxidative stress and mitochondrial disruption, leading to a mixed M1/M2 phenotype that boosts pro-fibrotic factor expression (12). Consequently, a positive feedback loop is established, amplifying fibrotic signaling.

#### 3.1.2 Platelet

During inflammatory and innate immune responses, there is a significant functional interaction between platelets and neutrophils. In the context of pulmonary diseases, this interaction is facilitated by various receptors, such as GPVI, TLR4 and Sema7A/PlexinC1 (76, 77), signaling pathways including NLRP6/PAR4 (78, 79), and secretory factors like CXCL5 and CXCL7 (80). These elements contribute to the accumulation of platelet-neutrophil complexes (PNCs) in the lungs and the formation of neutrophil extracellular traps (NETs), which can result in microvascular occlusion, NET-mediated tissue injury, and the amplification of inflammatory responses. Empirical studies have shown that in systemic sclerosis (SSc) and murine models of pulmonary fibrosis, platelet activation through the collagen receptor glycoprotein VI (GPVI) induces neutrophil activation and NET release, which ultimately promotes tissue fibrosis (81). Similarly, in the BLM-induced lung injury model, the administration of the platelet inhibitor Cangrelor has been shown to mitigate pulmonary neutrophil infiltration and subsequent fibrosis by inhibiting CD40–CD40L-mediated platelet-neutrophil adhesion (82).

#### 3.1.3 Complement system

The activation of the complement system plays a critical role in the recruitment and activation of neutrophils. In the context of COVID-19, the complement component C5a facilitates neutrophil infiltration across the vascular endothelium into tissues and enhances tissue factor expression, thereby exacerbating coagulation and inflammatory responses (83). The membrane attack complex (MAC/C5b-9) promotes the expression of neutrophil adhesion molecules on vascular endothelial cells, which aids in the formation of PNAs and accelerates neutrophil migration (84). Concurrent stimulation with C5a and anti-neutrophil cytoplasmic antibody (ANCA) can induce respiratory burst and degranulation in neutrophils (85). Additionally, C1q is capable of directly inducing NETosis in neutrophils primed with LPS (86). Activated neutrophils also modulate the complement system, creating an inflammatory amplification loop. For instance, neutrophil-secreted properdin significantly enhances the activation of the alternative pathway by stabilizing the C3 convertase and NETs carry complement components such as C3 and complement factor B (CFB) on their surfaces, which not only directly kill pathogens but also further promote local complement activation (87). Moreover, in the single-walled carbon nanotube (SWCNT) model, activation of the C5a-C5aR1 signaling pathway markedly enhances early neutrophil recruitment and the secretion of inflammatory cytokines, including TNF-α and IL-1β. The application of a C5aR1 antagonist effectively suppresses neutrophil-mediated early inflammatory responses and subsequent late-stage fibrosis (26).

#### 3.1.4 Lymphocyte

In the progression of pulmonary fibrosis, the interactions between neutrophils and lymphocytes are pivotal in regulating disease development. Neutrophils can establish a positive feedback loop with T cells, thereby exacerbating fibrosis. In the BLM model, Gr1+ neutrophils, activated by IL-1β and IL-17A signaling, produce B-cell activating factor (BAFF), which activates IL-17A+ T cells, further enhancing IL-17A expression and establishing a pro-fibrotic feedback loop (88). Ectopic colonization by the periodontitis pathogen Porphyromonas gingivalis facilitates the accumulation of neutrophils and Th17 cells in the lungs, where Th17 cells modulate neutrophil function through IL-17A secretion, thereby aggravating the fibrotic process (89). Furthermore, IL-17A+ γδ T cells can augment neutrophil infiltration and promote macrophage polarization toward the M2 phenotype in the lungs, accelerating fibrotic development (90). Conversely, B cells interact with senescent neutrophils via β integrins, facilitating their apoptosis and clearance, thus preventing abnormal neutrophil accumulation and suppressing inflammation and fibrosis (91). The administration of the CXCR4 antagonist AMD3100 correspondingly diminishes B lymphocyte accumulation and neutrophil infiltration in the lungs, thereby directly mitigating the progression of pulmonary fibrosis (91). Collectively, these mechanisms elucidate the bidirectional regulatory roles of neutrophil interactions with T and B lymphocytes in modulating the advancement of pulmonary fibrosis.

In conclusion, neutrophils function not solely as effector cells in pulmonary fibrosis but also as pivotal nodes within the immune interaction network. Their recruitment, activation, and the release of NETs are intricately regulated by macrophages, platelets, lymphocytes, and the complement system. Conversely, neutrophils and their NETs reciprocally influence the activities of macrophages and other immune cells, thereby establishing a complex positive feedback loop that ultimately facilitates the progression of pulmonary fibrosis.

### 3.2 Crosstalk between neutrophils and lung parenchymal cells

Neutrophils are integral to the pathogenesis of pulmonary fibrosis, engaging in intricate interactions with lung epithelial cells and fibroblasts. These interactions are predominantly facilitated by the release of NE and NETs, which contribute to epithelial injury, dysregulated repair processes, and fibroblast activation. Consequently, these processes lead to extracellular matrix deposition and the development of fibrosis.

#### 3.2.1 Epithelial cell

Neutrophil-derived proteases and NETs contribute directly to epithelial damage and phenotype transition. In BLM rats, neutrophil elastase promotes apoptosis of lung epithelial cells by activating caspase-3 and caspase-9 and inducing cytochrome c release. The inhibitor Sivelestat attenuates fibrosis by suppressing neutrophil chemotaxis and elastase-mediated apoptosis (92). In severe COVID-19, NETs are abundant in bronchoalveolar lavage fluid and cooperate with alveolar macrophage-derived factors (TGF-β, IL-8, IL-1β) to induce epithelial-mesenchymal transition (EMT) in alveolar epithelial cells. This is characterized by downregulation of E-cadherin and upregulation of α-SMA (93). A common transcriptomic signature in systemic lupus erythematosus (SLE) and COVID-19 patients also highlight NETs-induced EMT, suggesting a shared mechanism in fibrosis development (94). Furthermore, in a murine model of PM-induced fibrosis, neutrophil elastase released from accumulated neutrophils enhances EMT and fibrotic responses via macrophage-derived KC (73).

#### 3.2.2 Fibroblast

Neutrophils play a pivotal role in activating fibroblasts and facilitating their differentiation into matrix-producing myofibroblasts through various mechanisms, thereby contributing to fibrosis. Research shows that NE stimulates fibroblast proliferation by targeting insulin receptor substrate-1 (IRS-1) and induces fibroblast differentiation into myofibroblasts in a SMAD3-dependent yet transforming growth factor-beta (TGF-β)-independent manner, which is evidenced by the fact that treatment with the TGF-β receptor inhibitor SB431542 does not inhibit α-SMA production following NE exposure *in vitro (*95). Furthermore, both genetic deletion and pharmacological inhibition of NE have been shown to mitigate asbestos-induced pulmonary fibrosis in murine models (95). Additionally, components of NETs, such as DNA, histones, and myeloperoxidase, induce myofibroblast differentiation and collagen production in lung fibroblasts. IL-17 within NETs further enhances fibrotic responses by upregulating connective tissue growth factor (CCN2) (96). Furthermore, NETs activate lung fibroblasts through the TLR9–miR-7–SMAD2 axis in polymyositis-associated interstitial lung disease (97). Importantly, deficiency or inhibition of PAD4 suppresses NETs formation and significantly reduces bleomycin-induced fibrosis, highlighting the critical role of NETosis in fibrogenesis (98). From a therapeutic perspective, pharmacological inhibition of NET formation with pirfenidone has been shown to reduce fibroblast activation and NLRP3 inflammasome activity (99).

In summary, neutrophils promote pulmonary fibrosis through elastase-mediated epithelial apoptosis and NET-driven EMT and fibroblast activation. Targeting neutrophil-derived mediators may offer promising therapeutic strategies for attenuating fibrosis.

## 4 Neutrophils as prognostic biomarkers in pulmonary fibrosis

Accumulating clinical evidence highlights the pivotal role of neutrophil activity in prognosticating outcomes in pulmonary fibrosis. In patients with IPF, an elevated neutrophil-lymphocyte ratio (NLR) in peripheral blood is independently associated with reduced overall survival, thus serving as a robust hematological prognostic marker (100). Under conditions such as infection, chemotherapy, or tissue damage, G-CSF facilitates the mobilization of neutrophils from the bone marrow to the peripheral circulation. Notably, G-CSF levels in the bronchoalveolar lavage fluid (BALF) of IPF patients significantly exceed those in healthy controls and are predictive of survival rates, while inversely correlating with the decline in diffusing capacity of the lungs for carbon monoxide (DLCO) (101). Neutrophil percentages in BLAF bronchoalveolar lavage fluid (BALF) offer etiology-independent risk stratification, with each 10% increment associated with a 20% increase in mortality risk in antineutrophil cytoplasmic antibody-associated vasculitis interstitial lung disease (AAV-ILD) (hazard ratio [HR] = 1.195, 95% confidence interval [CI]: 1.018–1.404) (102). A threshold exceeding 6% predicts three-year mortality in progressive fibrosing interstitial lung disease (PF-ILD) with 79% sensitivity and 80% specificity (area under the curve [AUC] = 0.72) (103). Importantly, neutrophil effector molecules further refine prognostic accuracy. NETs in IPF lung tissue and BALF are associated with accelerated pulmonary function decline (P < 0.03) and independently predict mortality after multivariable adjustment (HR = 1.79–2.19) (104). This pattern is mirrored in post-COVID fibrosis models, where persistent NETosis at 30 days post-infection correlates with fibrotic severity (105). The complex formed by NE and α-1-antiprotease (α_-_-AP), referred to as the NE: α_-_-AP complex, when elevated, signifies increased NE release. Similarly, elevated levels of NE: α_-_-AP complex, in serum and BALF are correlated with clinical progression in general pulmonary fibrosis cohorts (106). Collectively, these findings underscore the potential of neutrophil-centric biomarkers in enhancing prognostic evaluation across various pulmonary conditions (**Table 2**).

**Table 2:** Prognostic neutrophil-related biomarkers in pulmonary fibrosis.

Biomarker	Sample source	Cohort/model	Prognostic significance	Ref.
NLR	Peripheral blood	IPF patients	Independent predictor of shorter overall survival	2022 (100)
G-CSF	BALF	IPF patients	1. Predicts survival rate 2. Positively correlates with DLCO decline	2022 (101)
Neutrophil percentage	BALF	AAV-ILD patients	Each 10% increase → 20% higher mortality risk	2023 (102)
neutrophil ratio	BALF	PF-ILD patients	>6% predicts 3-year mortality	2023 (103)
NETs	Lung tissue BALF	IPF patients	1. Correlates with pulmonary function decline. 2. Predicts reduced survival after multivariable adjustment.	2024 (104)
NETs	Lung tissue	Post-COVID murine model	Persistence for 30 days post-infection correlates with fibrosis severity.	2024 (105)
NE-α_-_-AP	Serum/BALF	Pulmonary fibrosis patients	Elevated NE-α_-_-AP complexes correlate with clinical severity.	1998 (106)

NLR, neutrophil-lymphocyte ratio; IPF, idiopathic pulmonary fibrosis; G-CSF, granulocyte colony-stimulating factor; BALF, bronchoalveolar lavage fluid; DLco, diffusing capacity of the lung for carbon monoxide; ILD, interstitial lung disease; AVV, antineutrophil cytoplasmic antibody-associated vasculitis; COVID, Corona virus disease; NETs, neutrophil extracellular traps; NE, neutrophil elastase; α_-_-AP, α-1-antiprotease; Ref., reference.

## 5 Therapeutic targeting: translating mechanisms to therapies

### 5.1 Targeting neutrophils in pulmonary fibrosis: specific approaches

Targeting neutrophil-mediated inflammatory responses has emerged as a promising therapeutic strategy for mitigating pulmonary fibrosis (**Table 3**). Inhibition of NE has shown efficacy across multiple models. In irradiated and LPS-challenged mice, administration of a neutrophil elastase inhibitor reduced neutrophil accumulation in BALF, suppressed TGF-β1 activation, and decreased phospho-SMAD2/3 expression, thereby protecting against fibrosis (107). Similarly, in BLM rats, the NE inhibitor Sivelestat attenuated fibrosis by blocking neutrophil chemotaxis via reduction of cytokine-induced neutrophil chemoattractant (CINC)-1 and inhibiting NE-induced lung cell apoptosis, mediated through suppression of caspase-3, caspase-9, and cytochrome c release (92). Sivelestat also alleviated bleomycin-induced pulmonary fibrosis in mice by inhibiting TGF-β activation and inflammatory cell recruitment, without affecting total TGF-β levels (108). Furthermore, in an asbestos-induced model, genetic deficiency or pharmacologic inhibition of NE with ONO-5046 reduced fibroblast proliferation, myofibroblast differentiation, and collagen deposition, independently of TGF-β (95).

**Table 3:** Therapeutic strategies targeting neutrophils in pulmonary fibrosis.

Target	Drug/intervention	Model	Mechanism	Ref.
NE	NE inhibitor (unspecified)	sublethal irradiation + LPS induced mice	Reduced neutrophil accumulation in BALF, inhibited TGF-β1 activation and phospho-SMAD2/3 expression.	2012 (107)
NE	Sivelestat	BLM-induced rats	Suppressed neutrophil chemotaxis (via CINC-1 inhibition) and inhibited NE-induced lung cell apoptosis (inhibited caspase-3/-9 activity and cytochrome c release).	2009 (92)
NE	Sivelestat	BLM-induced mice	Alleviated fibrosis via inhibition of TGF-β activation (reduced active TGF-β1, p-Smad2) and inflammatory cell recruitment. Did not significantly decrease total TGF-β1 levels.	2012 (108)
NE	ONO-5046	Asbestos-induced mice	Directly inhibited lung fibroblast proliferation and myofibroblast differentiation (in a TGF-β-independent fashion).	2015 (95)
NETosis PAD4	Cl-amidine (pan-PAD inhibitor)	BLM-induced mice	Inhibited PAD4 enzyme activity, reduced NETs formation, thereby alleviating inflammatory and fibrotic gene expression. Effect linked to PAD4 in hematopoietic cells.	2020 (98)
NETosis PAD4	Chloro-amidine	BLM-induced mice	Inhibited NETosis, improved lung function, reduced collagen deposition, potentially modulating Del-1 and p53 pathways.	2024 (109)
miR-155 NETs	Cap	BLM-induced mice	Downregulated miR-155-5p, reducing IL-1β, TNF-α, TGF-β1, consequently inhibiting NET production (reduced NE, PAD-4 levels).	2024 (113)
NETs	Conjugated linoleic acid	BLM-induced mice	Abrogated NET-induced M1/M2 macrophage polarization, oxidative stress, mitochondrial membrane disruption, and pro-fibrotic cytokine release.	2025 (12)
NETs	Pirfenidone	MAILD model	Inhibited NETs formation and NLRP3 inflammasome activation, attenuated EMT.	2025 (99)
C5a-C5aR1 signaling	PMX205 (C5aR1 antagonist)	SWCNT-induced mice	Inhibited C5a-C5aR1 axis, reducing early neutrophil recruitment and TNF-α/IL-1β secretion.	2025 (26)
Platelet-neutrophil interaction (CD40-CD40L)	Cangrelor	BLM-induced mice	Inhibited platelet activation, reducing neutrophil infiltration mediated by CD40-CD40L interaction.	2020 (82)
Multiple: NETs + NE	DNase-I@PDA NPs + Siv@PLGA NPs	LPS-induced mice Neutrophils from COVID-19 patients	Sequential nanotherapy: DNase-I degrades NETs, Sivelestat inhibits NE activity and neutrophil hyperactivation.	2025 (114)
Multiple (Cell Therapy)	GMSCs	BLM-induced mice	Reduced deleterious neutrophil accumulation, decreased release of NE, MMP-9, LPA, APL1, and TGF-β.	2021 (115)
Multiple: TGF-β + PD-1/ROS-NETs	JS-201	Lewis lung cancer model + radiation therapy	Reduced fibroblast proliferation by inhibiting TGF-β/SMAD pathway and ROS-mediated NETs release.	2025 (116)

NETs, neutrophil extracellular traps; NE, neutrophil elastase; BLM, bleomycin; BALF, bronchoalveolar lavage fluid; LPS, Lipopolysaccharide; TGF-β1, transforming growth factor-beta 1; CINC-1, cytokine-induced neutrophil chemoattractant 1; PAD4, peptidyl arginine deiminase 4; Del-1, developmental endothelial locus-1; TNF-α, tumor necrosis factor-alpha; IL-1β, interleukin-1 beta; Cap, Capsaicin; MAILD, murine myositis-associated interstitial lung disease; EMT, epithelial-mesenchymal transition; NLRP3, NOD-like receptor family, pyrin domain containing 3; C5a-C5aR1, complement component 5a - c5a receptor 1; SWCNT, single-walled carbon nanotube; CD40-CD40L, CD40 - CD40 ligand; COVID, corona virus disease; GMSCs, gingiva-derived mesenchymal stem cells; MMP-9, matrix metalloproteinase-9; LPA, lysophosphatidic acid; APL1, lysophosphatidic acid receptor 1; PD-1, programmed cell death protein 1; ROS, reactive oxygen species; Ref., reference.

Targeting NETs represents another viable approach. In BLM-induced fibrosis, inhibition of PAD4 using Cl-amidine or genetic knockout suppressed NETosis, reduced inflammatory and fibrotic gene expression, and ameliorated fibrosis. This effect was specifically linked to PAD4 expression in hematopoietic cells (98). Similarly, chloro-amidine inhibited NETosis *in vitro* and *in vivo*, improving lung function, reducing collagen deposition, and modulating Del-1 and p53 pathways (109). However, PAD4 inhibition presents risks: it can suppress virus-specific CD8^+^ T cell responses in SARS-CoV-2 models, affecting adaptive immunity (110). PAD2 may compensate for PAD4 in conditions like Kawasaki disease, and inhibitors like Cl-amidine might act on non-PAD4 targets, leading to incomplete effects or side effects (111). Timing is crucial; in myocardial infarction, early PAD4 inhibition worsens injury, while delayed treatment improves outcomes (112). Thus, while PAD4 targeting could aid pulmonary fibrosis treatment, it risks T-cell suppression, off-target effects, and requires precise timing and so on. Additionally, in BLM model, miR-155 inhibition by Capsaicin reduced NET production via downregulation of IL-1β, TNF-α, and TGF-β1, decreasing levels of NE, PAD4, and hydroxyproline (113). Conjugated linoleic acid CLA also abrogated NET-induced M1 and M2 macrophage polarization and pro-fibrotic cytokine release *in vitro (*26). Further, pirfenidone reduced NET formation, suppressed NLRP3 inflammasome activation, and attenuated EMT in a myositis-associated ILD model (99).

Combination strategies targeting multiple neutrophil-derived components have enhanced efficacy. In a LPS-induced model, sequential delivery of DNase-I (to digest NETs) and sivelestat (to inhibit NE) via nanoparticles reduced fibrosis, improved lung function, and decreased NET biomarkers in neutrophils from COVID-19 patients (114). Beyond direct neutrophil targeting, interrupting neutrophil-platelet interactions via the CD40–CD40L axis with cangrelor reduced neutrophil infiltration and attenuated fibrosis (82).

Other strategies include modulating neutrophil recruitment pathways. Inhibition of C5a–C5aR1 signaling with PMX205 reduced early neutrophil recruitment and pro-inflammatory cytokines (TNF-α, IL-1β), thereby mitigating inflammation and fibrosis in a model of SWCNT-induced lung injury (26). Cellular therapies, such as gingiva-derived mesenchymal stem cells (GMSCs), reduced neutrophil accumulation and decreased levels of NE, MMP-9, lysophosphatidic acid (LPA), lysophosphatidic acid receptor 1(APL1), and TGF-β in bleomycin-induced fibrosis (115). Finally, dual targeting of PD-1 and TGF-β signaling with JS-201 suppressed radiation-induced fibrosis by inhibiting fibroblast proliferation and NETosis mediated by ROS (116).

In summary, therapeutic interventions have focused on inhibiting neutrophil recruitment, neutralizing cytotoxic enzymes, preventing NETs formation, or dismantling existing NETs. These approaches collectively underscore the centrality of neutrophils in fibrogenesis of lungs and highlight multiple translational opportunities.

### 5.2 Neutrophil-targeted therapy as a pan-fibrotic strategy: prospects and challenges

Neutrophils has been recognized as pivotal contributors to tissue fibrosis across various organ-specific fibrotic diseases. Nevertheless, the distinct characteristics of organ microenvironments and the progression stages of these diseases present both opportunities and challenges for the development of therapeutic strategies targeting these cells, which hold potential for broad-spectrum anti-fibrotic applications.

In the context of renal fibrosis, through methods such as PAD4 deletion or DNase treatment, markedly mitigates renal fibrosis in the UUO model, and then the introduction of exogenous NETs has been shown to exacerbate the pathological condition (117). Additionally, a subset of neutrophils characterized by siglec-F+ expression has been identified as highly expressing pro-fibrotic factors, and the transplantation of these cells has been observed to promote the progression of fibrosis. This observation is consistent across both lung and renal fibrosis, suggesting the presence of a conserved fibrotic mechanism across different organs (118).

In the context of hepatic fibrosis, neutrophil function exhibits considerable context-dependency. In models of alcohol-associated MASH, NETs and their associated enzymes, NE and proteinase 3, play a direct role in driving the fibrotic process (119, 120). Conversely, in chronic liver injury induced by carbon tetrachloride (CCl4), NETs mediated by PAD4 exert a limited influence on the extent of fibrosis, suggesting the involvement of alternative activation pathways (62). Furthermore, during the resolution phase of inflammation, neutrophils can facilitate the transition of macrophages to a pro-repair phenotype through mediators such as microRNA-223, thereby exerting an anti-fibrotic effect (121).

In the context of cardiac fibrosis, the functionality of neutrophils exhibits a distinct temporal dependency. Post-acute myocardial infarction (MI), neutrophils play a crucial role in facilitating reparative fibrosis during the initial stages (122). Conversely, their sustained activation in the chronic phase, notably through mechanisms such as NET-induced pyroptosis and the subsequent fibrotic responses in cardiac fibroblasts, contributes to pathological remodeling and the progression to heart failure (123).

Neutrophils are crucial in linking chronic injuries to fibrosis, making them a promising target for fibrotic therapy, particularly by inhibiting NET formation. However, two main challenges exist: their dual roles in different organs, such as the liver, and the timing of intervention, as seen in cardiac repair where early inhibition might hinder healing. Future research should aim to identify specific neutrophil functions and molecules across contexts to develop precise, targeted treatments.

## 6 Concluding remarks and future perspectives

Neutrophils, as pivotal effector cells within the innate immune system, have been definitively identified as essential contributors to the pathogenesis of pulmonary fibrosis. They play a direct role in promoting inflammation, tissue damage, and the irreversible fibrotic cascade through mechanisms such as tissue infiltration, the release of NE, and the formation of NETs. The intricate interactive network among neutrophils, alveolar macrophages, epithelial cells, and fibroblasts exacerbates and sustains a deleterious cycle of “inflammation-damage-fibrosis,” thereby complicating therapeutic interventions. Clinical data suggest that neutrophil counts and NET-associated components are valuable biomarkers for disease evaluation and prognosis. Notably, therapeutic approaches targeting neutrophil-mediated pathways, including NE inhibition, PAD4 blockade, or disruption of the C5a-C5aR1 axis, have shown significant anti-fibrotic effects in preclinical models, underscoring their potential as innovative therapeutic targets.

Future research should strategically concentrate on several critical areas. Firstly, it is imperative to elucidate the dynamic functional roles of neutrophils and their upstream regulatory networks across various disease stages to facilitate stage-specific interventions. Secondly, a comprehensive understanding of the neutrophil’s central role within the immune-stromal-epithelial axis is required, with particular emphasis on how their interactions with adaptive immune cells influence the fibrotic environment. From a therapeutic standpoint, efforts must be intensified to expedite the translation of targeted agents from bench to bedside and to investigate the potential synergistic effects of combining these novel therapies with existing anti-fibrotic drugs.

In conclusion, neutrophils represent a crucial focal point in the understanding and treatment of pulmonary fibrosis. Future investigations should not only enhance our mechanistic insights but also prioritize the development of robust translational pathways to the clinic, ultimately providing new hope for patients.

## Glossary

PF: pulmonary fibrosis
ECM: excessive extracellular matrix
ILD: interstitial lung disease
IPF: particularly idiopathic pulmonary fibrosis
NETs: neutrophil extracellular traps
TGF-β1: transforming growth factor-beta 1
NLR: neutrophil-lymphocyte ratio
C5aR1: complement component 5a receptor 1
CXCL14: C-X-C motif chemokine ligand 14
IL-36γ: interleukin-36 gamma
COPD: chronic obstructive pulmonary disease
fMLP: N-formylmethionyl-leucyl-phenylalanine
G-CSF: granulocyte colony-stimulating factor
LRG1: leucine-rich alpha-2-glycoprotein 1
FPR1: formyl peptide receptor 1
LPS: lipopolysaccharide
ROS: reactive oxygen species
NADPH: nicotinamide adenine dinucleotide phosphate
PSM: phenol-soluble modulin
FPR2: formyl peptide receptor 2
cGAS-STING: cyclic GMP-AMP synthase- stimulator of interferon genes
CR3: complement receptor 3
MPO: myeloperoxidase
NE: neutrophil elastase
NGAL: neutrophil gelatinase-associated lipocalin
MMP: matrix metalloproteinase
ERK1/2: extracellular signal-regulated kinase 1/2
CF: cystic fibrosis
NLRP3: NOD-like receptor family, pyrin domain containing 3
COVID: corona virus disease
IL-1β: interleukin-1 beta
IL-18: interleukin-18
TGF-β: transforming growth factor-beta
KC: keratinocyte chemoattractant
CXCL1: C-X-C motif chemokine ligand 1
PM: particulate matter
VIP: vasoactive intestinal peptide
CXCL2: C-X-C motif chemokine ligand 2
THP-1: human acute monocytic leukemia cell line
HOCl: hypochlorous acid
SSc: systemic sclerosis
GPVI: glycoprotein VI
CD40-CD40L: CD40 - CD40 ligand
BALF: bronchoalveolar lavage fluid
IL-17A: interleukin-17A
SWCNT: single-walled carbon nanotube
C5a-C5aR1: complement component 5a - c5a receptor 1
TNF-α: tumor necrosis factor-alpha
BBP: benzyl butyl phthalate
BLM: bleomycin
α-SMA: alpha-smooth muscle actin
EMT: epithelial-mesenchymal transition
AM: alveolar macrophage
SLE: systemic lupus erythematosus
NSIP: Nonspecific Interstitial Pneumonia
CNN2: calponin 2
PAD4: peptidyl arginine deiminase 4
TLR9: toll-like receptor 9
MAILD: murine myositis-associated interstitial lung disease
CINC-1: cytokine-induced neutrophil chemoattractant 1
Del-1: developmental endothelial locus-1
Cap: Capsaicin
GMSCs: gingiva-derived mesenchymal stem cells
MMP-9: matrix metalloproteinase-9
LPA: lysophosphatidic acid
APL1: lysophosphatidic acid receptor 1
PD-1: programmed cell death protein 1.
