# Correction: Neutrophils as key drivers of pulmonary fibrosis: unveiling mechanisms and therapeutic implications

**DOI:** 10.3389/fimmu.2025.1761117

**Published:** 2026-01-02

**Authors:** Xiuping Liang, Yanhong Li, Ziyi Tang, Yubin Luo, Yi Liu

**Affiliations:** 1 Department of Rheumatology & Immunology, Laboratory of Rheumatology and Immunology, West China Hospital, Sichuan University, Chengdu, Sichuan, China; 2 West China Lecheng Hospital, Sichuan University, Boao, Hainan, China

**Keywords:** pulmonary fibrosis, neutrophils, neutrophil extracellular traps, neutrophil elastase, crosstalk

There was a mistake in **Figure 1** and **Figure 2** as published. The content of **Figure 1** and **Figure 2** were erroneously switched. The corrected **Figure 1** and **Figure 2** appears below.

**Figure 1:**
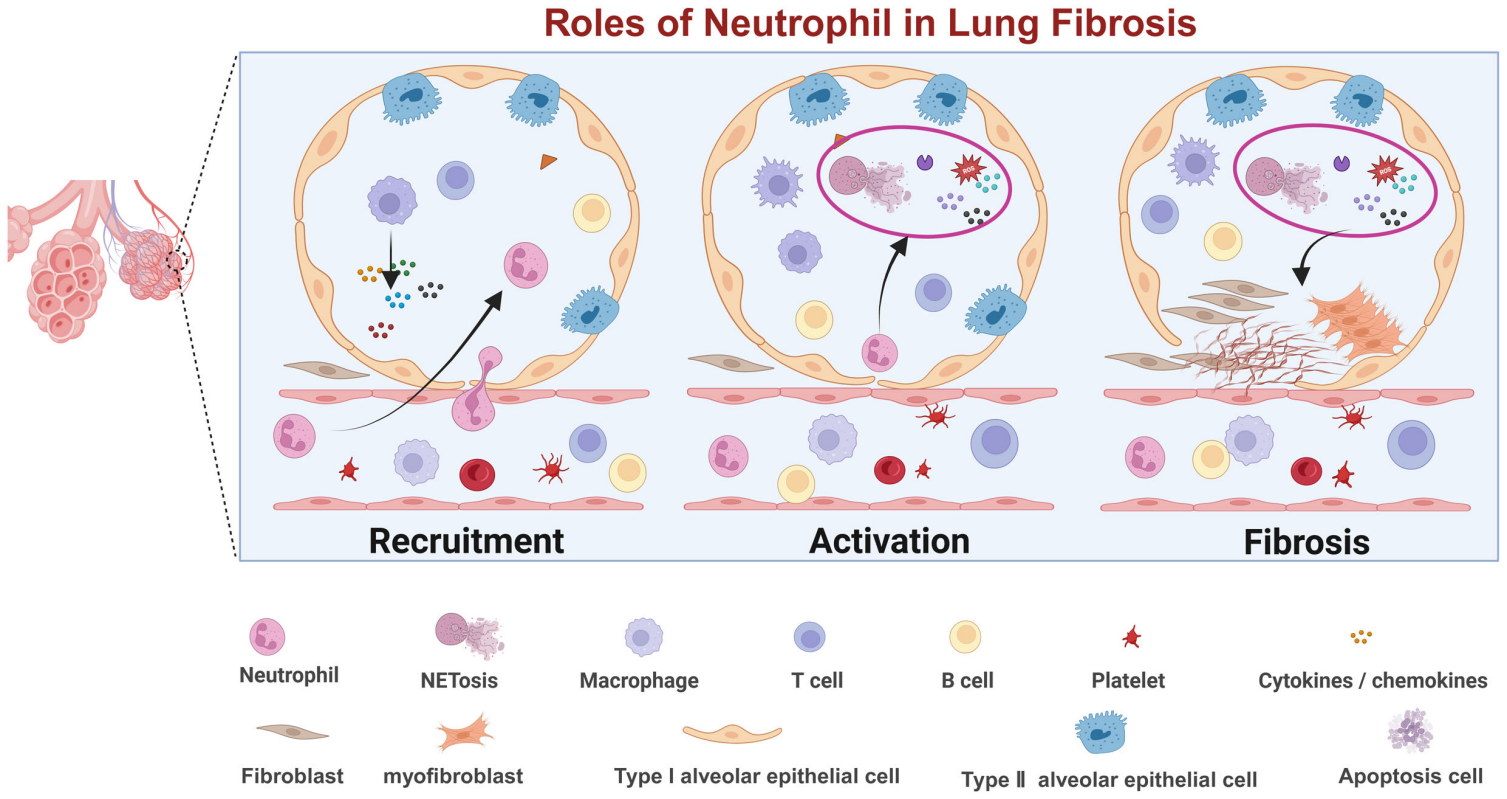
Diagram illustrating the roles of neutrophils in lung fibrosis across three stages: recruitment, activation, and fibrosis.

**Figure 2:**
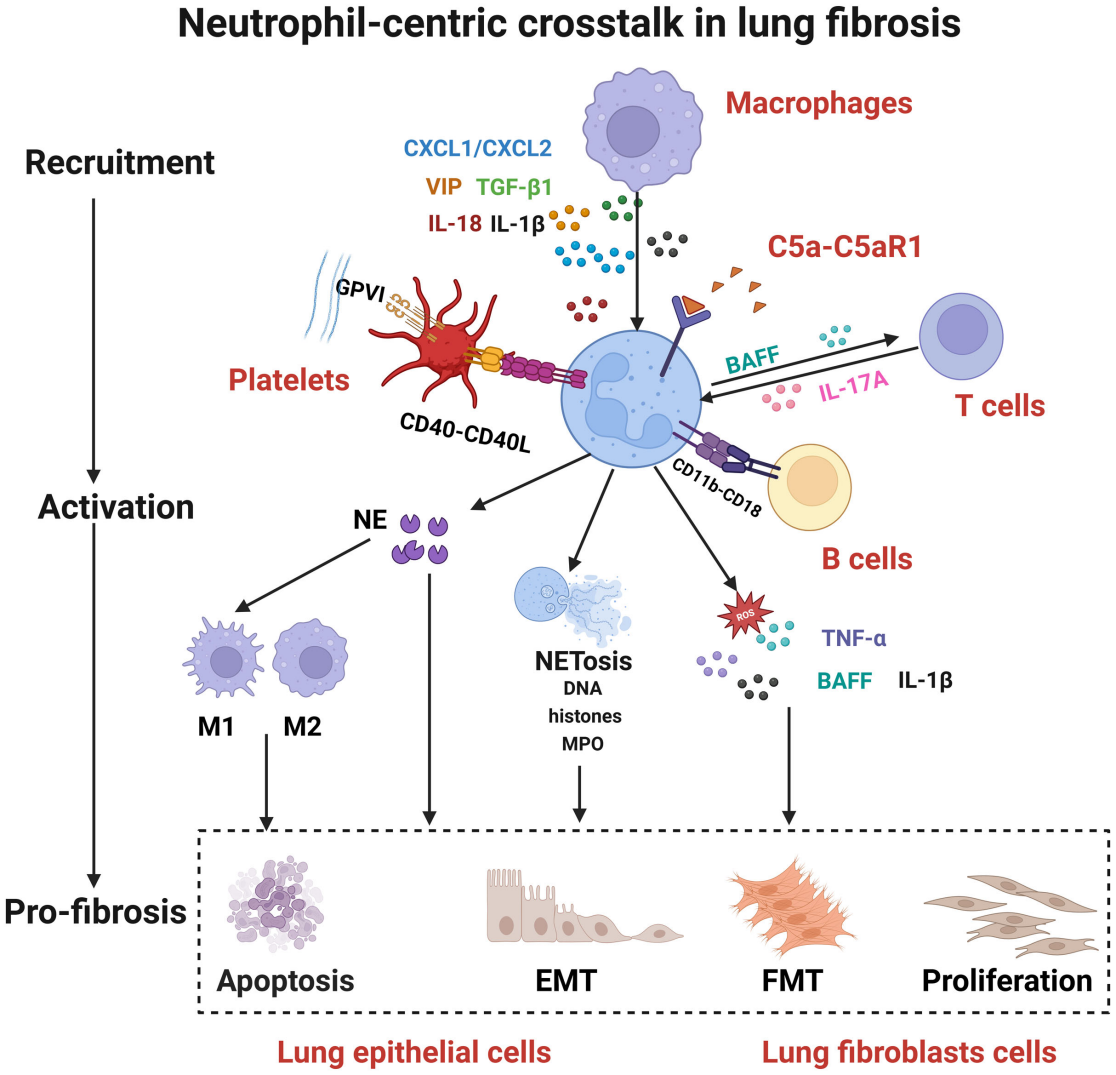
Neutrophil-centric crosstalk in lung fibrosis. The fibrotic process involves coordinated cellular interactions: (1) Macrophages, T cells, B cells and platelets recruit and activate neutrophils; (2) Activated neutrophils release NETs, NE, IL-1β, and TNF-α; (3) These effector molecules target lung epithelial and fibroblast cells, promoting apoptosis, EMT, and FMT to drive fibrosis. CXCL2, C-X-C motif chemokine ligand 2; VIP, vasoactive intestinal peptide; TGF-β1, transforming growth factor beta 1; IL-18, interleukin-18; IL-1β, interleukin-1 beta; C5a, complement component 5a; C5aR1, complement C5a receptor 1; GPV, glycoprotein V; CD40L, CD40 ligand; BAFF, B cell activating factor; IL-17A, interleukin-17A; NETosis, neutrophil extracellular trap formation; DNA, deoxyribonucleic acid; TNF-α, tumor necrosis factor alpha; IL-1β, interleukin-1 beta; MPO, myeloperoxidase; NE, neutrophil elastase; EMT, epithelial-mesenchymal transition; FMT, fibroblast-myofibroblasttransition.

The original version of this article has been updated.

